# Some Pancreatic Lesions and Their Clinical Aspects

**Published:** 1904-06-11

**Authors:** 


### SOME PANCREATIC LESIONS AND THEIR CLINICAL ASPECTS.

Despite the careful and illuminating inquiries which of late have been directed towards the physiological and pathological phenomena connected with the pancreas, the clinician is still in most cases far from being able to form an accurate and reliable opinion as to the state of this organ when the question of its condition arises. The reasons for this failure are to be found in the comparative rarity of pancreatic disease, the inaccessibility of the gland to palpation save in exceptional cases, the multiplicity of its functions, the absence of well-marked physical signs, and the fact that lesions of the pancreas are usually secondary to changes in other organs, *e.g.* the duodenum and bile passages, a circumstance which may cause symptoms that serve to divert attention from the pancreas. As Dr. Eugene Opie points out, the knowledge that certain lesions of the pancreas are frequently found in association with more readily recognised affections of neighbouring structures should help rather than hinder the clinician. This writer separates diseases of the pancreas, for purposes of convenience, into (1) those which affect the secreting apparatus of the gland, and (2) those which have their seat in the islands of Langerhans and interfere with the influence which these structures exert on carbohydrate metabolism. He lays much emphasis on the importance of bearing in mind the histological and gross anatomy of the gland and its physiological functions in order to fully appreciate the alterations which may result from disease in this region. Thus he refers to Pawlow's observation that the pancreatic juice obtained direct from the gland through a fistula is capable of only weak solvent action upon proteid material, whereas, after it has come in contact with the succus entericus, or with bile, the proteolytic power of the pancreatic ferment becomes enormously increased. Again, it has been shown that the efficiency of the fat-splitting ferment is doubled or trebled in the presence of bile. These two facts have an important bearing in connection with acute hæmorrhagic pancreatitis and fat necrosis. It has been shown that fat necrosis is due to the presence in the affected areas of fat-splitting ferment derived from the pancreas, while hæmorrhagic pancreatitis is due to auto-digestion of the gland made possible, in some cases, by the presence in it of bile, which is capable of increasing the proteolytic power of the pancreatic ferment. This theory is supported by the fact that in a considerable proportion of the cases recorded in the literature of the subject calculi have been found in the gall-bladder or bile ducts. In four out of five cases which have come under Dr. Opie's personal observation, gall-stones have been present, and there is good evidence to support the theory that acute pancreatitis is caused by the blocking of the common biliary and pancreatic duct, and the consequent passage of bile into the pancreas. Dr. Opie explains the comparative rarity of acute hæmorrhagic pancreatitis occurring in gall-stone cases by the circumstance that a stone lodged in the ampulla of Vater while occluding the duodenal orifice must not be so large as to block the biliary and pancreatic ducts, and he notes in this connection that in all the cases of acute pancreatitis which he has examined, the gall-stones present in the bile passages and in the gall-bladder have been of very small size. Again, in a considerable proportion of individuals the anatomical conformation of the ampulla of Vater is such as to preclude the possibility of a stone being lodged in the common duct without occluding also the pancreatic and bile ducts; while in yet other cases the biliary and pancreatic ducts open by separate orifices into the duodenum. On the basis of these observations it is not difficult to explain the rarity of acute pancreatitis, assuming that its most frequent cause is the lodgement of a gall-stone in the ampulla of Vater.

The symptoms of this disorder are those of an acute abdominal lesion. The localisation of the pain in the epigastrium and the severity of the symptoms being the most characteristic features, while the discovery of fat necrosis during an exploratory laparotomy will indicate the source of trouble.

The clinical aspect of the facts above-mentioned are: (1) The presence of jaundice, or the history of previous attacks of gall-stone colic may give confirmation to the diagnosis of acute hæmorrhagic pancreatitis, when somewhat indefinite symptoms suggest the presence of this condition; (2) when during an operation undertaken for the relief of obscure abdominal symptoms the existence of acute hæmorrhagic pancreatitis is indicated by the presence of fat necrosis, examination of the gall-bladder and bile passages will in a considerable proportion of cases reveal the presence of gall-stones, the removal of which offers the best chance of recovery.

Another lesion which has its origin in the secreting apparatus of the gland is the chronic inflammation which follows obstruction or ascending infection of the pancreatic ducts. The usual causes of obstruction of the larger pancreatic duct are biliary calculi, pancreatic calculi, and carcinoma. Induration of the head of the pancreas due to chronic inflammation is not infrequently found in gall-stone cases. Although this affection is not usually considered to give rise to any characteristic symptoms, it has been thought by Mayo Robson to be the cause of severe pain in the epigastrium and in the midscapular region, vomiting, loss of weight and strength, and even death. Dr. Opie remarks that it is but seldom that the presence of fatty stools serve to aid the diagnosis of pancreatic disease.

It is of importance to remember the occurrence of chronic inflammation of the head of the pancreas, otherwise the discovery of an indurated tumour in this region in the course of an operation for supposed gall-stones may lead to a mistaken diagnosis of cancer.

